# Investigation of Precise Molecular Mechanistic Action of Tobacco-Associated Carcinogen ‘NNK’ Induced Carcinogenesis: A System Biology Approach

**DOI:** 10.3390/genes10080564

**Published:** 2019-07-26

**Authors:** Anukriti, Anupam Dhasmana, Swati Uniyal, Pallavi Somvanshi, Uma Bhardwaj, Meenu Gupta, Shafiul Haque, Mohtashim Lohani, Dhruv Kumar, Janne Ruokolainen, Kavindra Kumar Kesari

**Affiliations:** 1 Department of Biosciences, Himalayan Institute of Medical Sciences, Swami Rama Himalayan University, Dehradun 248016, India; 2 School of Biotechnology, Gautam Buddha University, Greater Noida 201310, India; 3 Department of Biotechnology, TERI School of Advanced Studies, 10, Institutional Area, Vasant Kunj, New Delhi 110070, India; 4 Research and PhD Cell, Swami Rama Himalayan University, Dehradun 248016, India; 5 Department of Radiotherapy, Cancer Research Institute, Himalayan Institute of Medical Sciences, Swami Rama Himalayan University, Dehradun 248016, India; 6 Research and Scientific Studies Unit, College of Nursing and Allied Health Sciences, Jazan University, Jazan 45142, Saudi Arabia; 7 Department of EMS, College of Applied Medical Sciences, Jazan University, Jazan 45142, Saudi Arabia; 8 Amity Institute of Molecular Medicine & Stem Cell Research, Amity University, Noida, Uttar Pradesh 201303, India; 9 Department of Applied Physics, Aalto University, 00076 Espoo, Finland

**Keywords:** NNK, cancer, systems biology, protein–protein interaction network, topological analysis, gene ontology

## Abstract

Cancer is the second deadliest disease listed by the WHO. One of the major causes of cancer disease is tobacco and consumption possibly due to its main component, 4-(methylnitrosamino)-1-(3-pyridyl)-1-butanone (NNK). A plethora of studies have been conducted in the past aiming to decipher the association of NNK with other diseases. However, it is strongly linked with cancer development. Despite these studies, a clear molecular mechanism and the impact of NNK on various system-level networks is not known. In the present study, system biology tools were employed to understand the key regulatory mechanisms and the perturbations that will happen in the cellular processes due to NNK. To investigate the system level influence of the carcinogen, NNK rewired protein–protein interaction network (PPIN) was generated from 544 reported proteins drawn out from 1317 articles retrieved from PubMed. The noise was removed from PPIN by the method of modulation. Gene ontology (GO) enrichment was performed on the seed proteins extracted from various modules to find the most affected pathways by the genes/proteins. For the modulation, Molecular COmplex DEtection (MCODE) was used to generate 19 modules containing 115 seed proteins. Further, scrutiny of the targeted biomolecules was done by the graph theory and molecular docking. GO enrichment analysis revealed that mostly cell cycle regulatory proteins were affected by NNK.

## 1. Introduction

Cancer is one of the major non-communicable diseases [1] and is accountable for millions of deaths per year worldwide. According to the World Health Organization (WHO), cancer is the second major cause of morbidity, with an estimate of 9.6 billion deaths in 2018 [2]. Cancer is a multistage process caused by aberrations in the cellular processes. Cancer is not only caused by mutation in any single gene but also by the accumulation of mutations in multiple genes, a phenomenon described as ‘oncogene addiction’ [3]. According to the WHO, there are mainly three reasons that lead to these aberrations, with tobacco consumption heading the list, which is single-handedly responsible for around 22% of deaths by cancer globally [4].

Currently, we have immense information on how tobacco consumption has direct implications in cancer, specially lung, head and neck, stomach, liver, and pancreatic cancers [5,6]. 4-(methylnitrosamino)-1-(3-pyridyl)-1-butanone (NNK) is one of the main components in tobacco that plays a major role in the causation of cancer [6]. NNK and its derivative, 4-(methylnitrosamino)-1-(3-pyridyl)-1-butanol (NNAL), binds with the DNA and forms DNA adducts, the resultant of which may lead to genetic mutations followed by the deregulation of normal cellular processes [6,7].

NNK is not just responsible for causing cancer but also holds serious implications in other diseases as well. Earlier studies have shown that NNK has significant impact on steatohepatitis [8], Alzheimer’s disease [9], and tuberculosis [10], for example.

In this study, attempts have been made to exploit the system biology approach for an investigation of the overall impact of tobacco-generated carcinogen NNK on molecular systems.

Systems biology is an interdisciplinary field, which is a combination of mathematics, computer science, and biology [11]. Systems biology holds importance as it helps in getting a holistic view of the connections of biomolecules. It provides an anti-reductionist approach towards the involvement of different biomolecular components in a variety of biological systems. It focuses on the development of interactome of the affected targets and then analyzing it by using mathematical models [12]. Graph theory is the core assessment tool for the topological analysis of the interactome that aims to identify hub biomolecular targets based on their clustering coefficients, degrees, and betweenness centralities. Whereas, clustering and gene ontology (GO) enrichment analysis categorize the biomolecules on the basis of functions to procure more promising insight into complex networks. This study aims to find the most probable biomolecular targets of tobacco-associated carcinogen NNK along with its interactions and associated pathways that get perturbated by various cellular mechanisms and lead to cancer development. The most probable key targets of NNK are identified on the basis of their bottleneck scores and based on their thermodynamic interactions with NNK calculated by molecular docking simulations.

## 2. Materials and Methods

**Figure 1:**
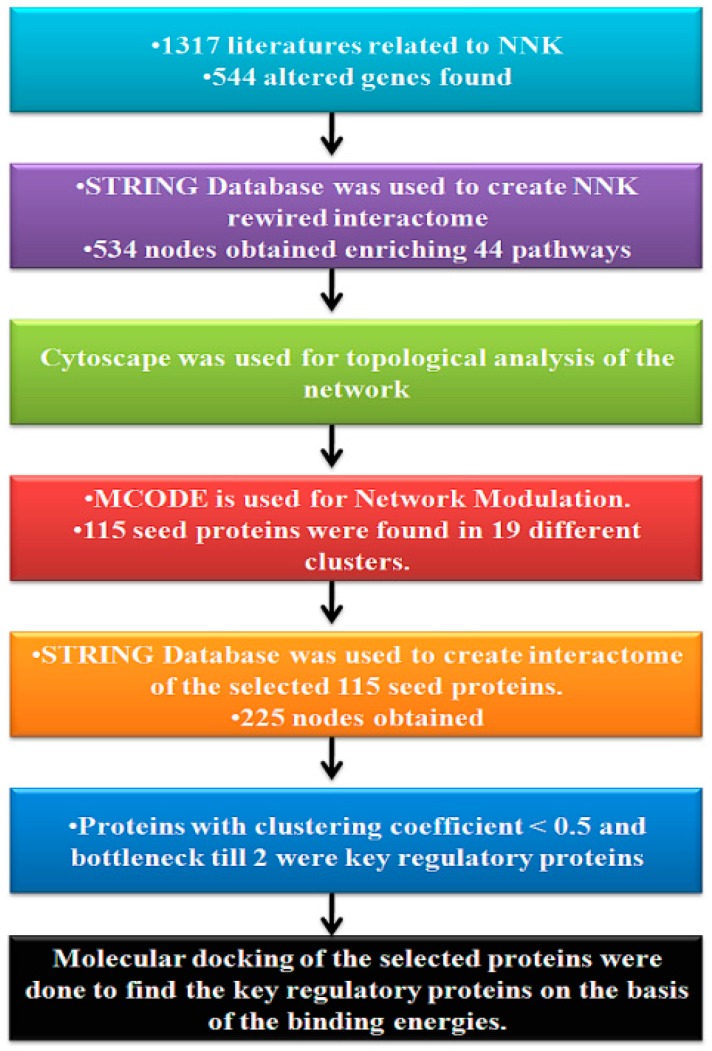
Schematic diagram of the adopted methodology.

The full methodology scheme is mentioned in Figure 1.

### 2.1. Construction and Visualization of Protein–Protein Interaction Network

In total, 544 biomolecular targets were found to be affected by NNK in approximately 1320 studies using PubMed. A protein–protein interaction network was developed using the STRING database version 10.5 [13]. The network was evidence-based and developed with the highest confidence level score of 0.9, having 50 interactors in the first as well as second shell.

### 2.2. Protein–Protein Interaction Network (PPIN) Analysis

The Cytoscape Software (version 3.6.1) program [14] was used for the analysis of the protein–protein interaction network (PPIN) to generate protein interaction networks. Network analyzer, an in-built plugin of Cytoscape, was used to analyze the topological properties of an NNK modulated PPIN [15]. The topological properties of any network provide a deep insight into complex biological networks [16,17]. The topological analysis also helps in reducing noise in the data and offers reliable information regarding the network [18]. Node properties, like degree distribution, shortest path length, average clustering coefficient, betweenness centrality, and closeness centrality, were also analyzed.

### 2.3. Protein Interaction Network Modular Analysis and Pathway Enrichment

Clusters or modules are closely connected nodes in a network that come together and form a dense sub-network [19]. The analysis of clusters or modules helps in attaining detailed information about PPIN. Molecular COmplex DEtection (MCODE) is a plug-in available in the Cytoscape software program, which was used for the cluster analysis. The clusters are scored on the basis of size and density—a high score means a big and dense cluster—while gene ontology (GO) serves the purpose of validating the cluster that belongs to a specific function [20]. Thus, the enrichment of clusters helps in enriching the pathways by providing an additional number of external genes that are not present in the dataset. GO analysis provides detailed information about the biological process underneath that cluster. For GO functional enrichment analysis, ClueGO (version 2.5.1) plug-in of Cytoscape software was used [21]. The analysis was done using a threshold *p* value < 0.05. A two-sided hypergeometric test was used for the statistical analysis along with the Bonferroni correction method, in case applied.

### 2.4. Molecular Docking Analysis

Molecular docking is one of the most preferred methods to find the orientation of two molecules when they form a complex. Docking simulation also explores the thermodynamic stability of the complexes by providing information regarding the binding energy and inhibition constant (K_i_) value and helps in finding the best binding modes or orientations of a ligand with its biomolecule. The docking parameters used were based on the studies published by [22]. Autodock 4.0 MGL suite [23] was used for docking simulations. The simulations were performed on AMD E1-6015 APU processor, CPU 1.4 GHz and 4 GB RAM of Hewlett-Packard (HP) machine.

## 3. Results

### 3.1. Construction of the Network

**Figure 2:**
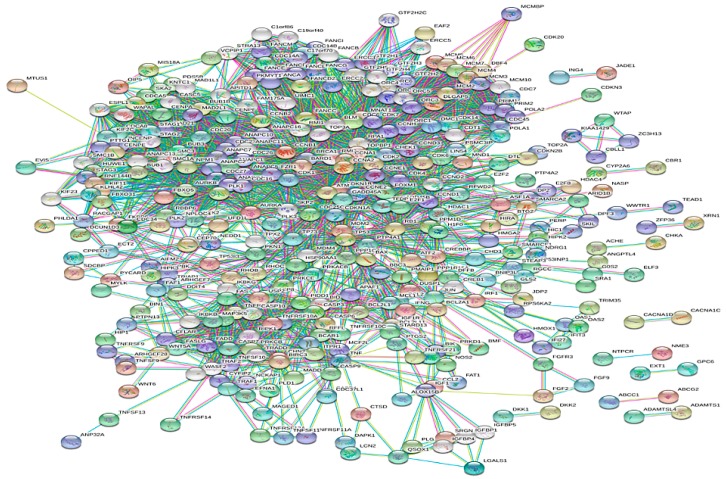
STRING generated NNK rewired protein–protein interaction network with 534 nodes and 2909 edges.

**Figure 3:**
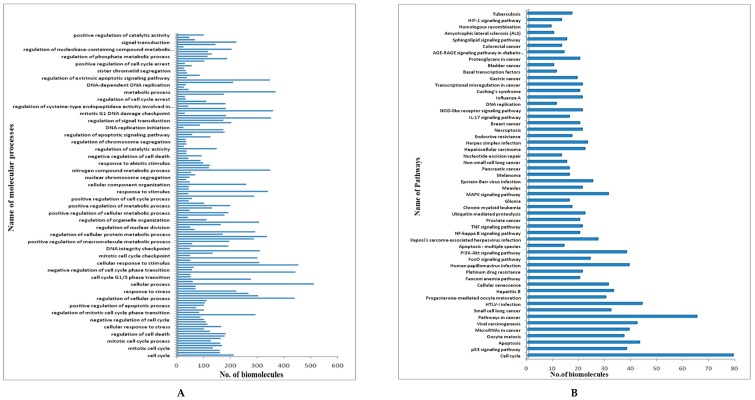
(**A**) Processes enriched by NNK rewired PPIN. (**B**) Pathways enriched by NNK rewired PPIN.

In total, 544 biomolecular targets were found to be affected by NNK interaction through a literature survey. A protein–protein interaction network (PPIN) was developed using the STRING database version 10.5. The network developed at the 0.9 confidence level score and 50–50 interactors in the first and second shell comprised of 534 nodes and 2909 edges. The average node degree was 10.09 and average local clustering coefficient was 0.501. The PPI enrichment *p* value was <1 × 10^−16^. Figure 2 represents the NNK rewired protein–protein interaction network having 534 nodes and 2909 edges. Figure 3A,B depicts the number of biomolecular targets involved in various processes and pathways, respectively.

### 3.2. Topological Properties of the Network

**Figure 4:**
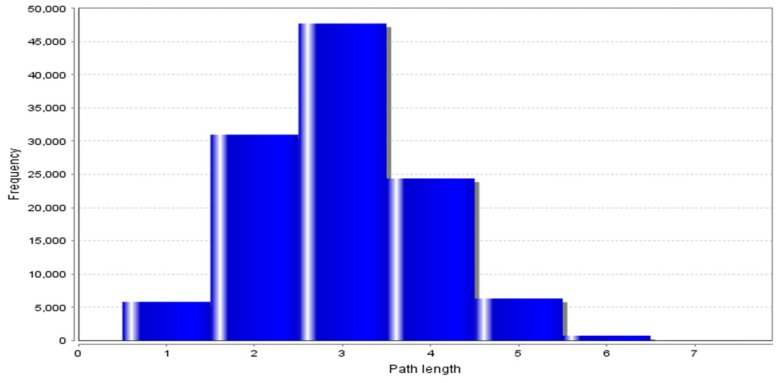
Characteristic path length distribution: 2971.

**Figure 5:**
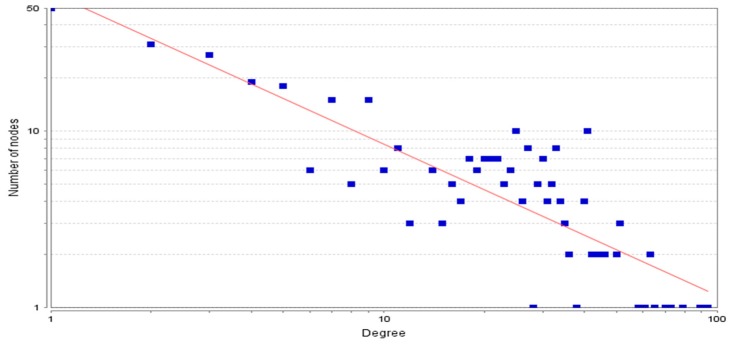
Node degree distribution following power law fitting y = 61,323x^−0.861^ (R-squared 0.722).

**Figure 6:**
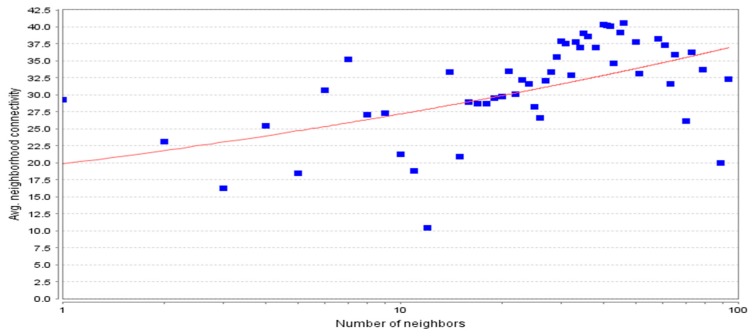
Average (Avg.) neighborhood connectivity distribution following power law fitting y = 19,838x^0.137^ (R-squared 0.253). The average number of neighbors is 15,940.

**Figure 7:**
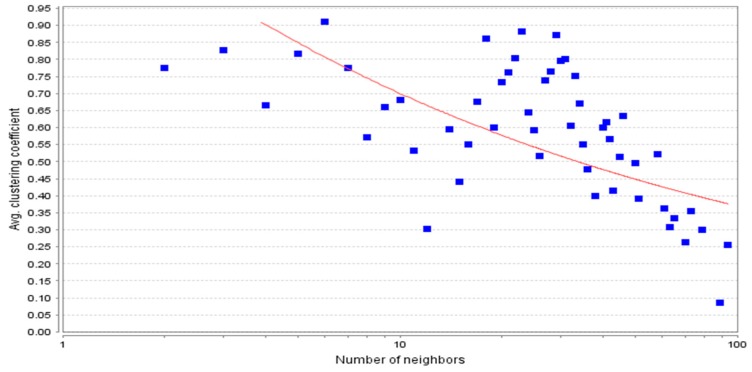
Average (Avg.) clustering coefficient following power law fitting y = 1.326 x^−0.277^ (R-squared 0.329). Clustering coefficient distribution of 0.597.

The protein–protein interaction network developed was further analyzed using Cytoscape software. The topological properties of the PPIN calculated with the help of Network Analyzer plug-in were the shortest path length average, neighborhood connectivity distribution, clustering coefficient, node degree distribution, betweenness centrality, closeness centrality, etc. The shortest path length in any network depicts the shortest communication mode between two nodes. Figure 4 shows a graphical representation of path length distribution 2971. This means that at the 2971-unit path length, the information is being passed on at the highest frequency. The degree of a node describes the connectivity of a node with other nodes; it is the total number of links, which are either reaching or starting from that node [24]. The node degree distribution (Figure 5) is one of the most important topological properties of a network. It fits a power law that indicates the presence of hubs in the network [25]. The nodes, which lie close to the power line and have a higher degree, can be considered as the hubs. The average neighborhood connectivity distribution stands for the average number of neighbors, which was observed as 15,940 in this study (Figure 6). Figure 6 shows the average connections of each node with its neighbors. The above parameter helps in understanding the density of a network. The clustering coefficient of a network depicts the tendency of a graph to be divided into clusters [26]. The local clustering coefficient is the number of edges around a particular node, whereas the average clustering coefficient is the clustering coefficient of the whole network [25], and in this study, the average clustering coefficient in this network was found to be 0.597 (Figure 7).

### 3.3. Clustering and GO Enrichment Analysis

**Figure 8:**
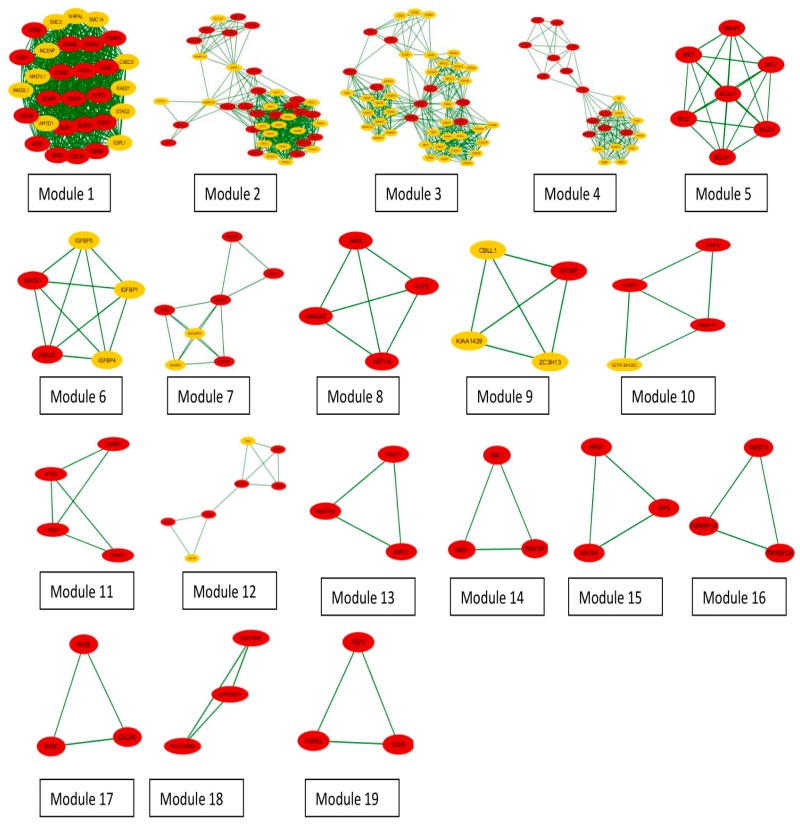
Network modules: red circles show the seed proteins and yellow circles are the connectors.

For modular analysis and pathway enrichment, the MCODE plug-in of the Cytoscape was used. Modules or clusters were created from the network and were scored on the basis of their size and density. A high score depicts a denser and tighter cluster. Formation of clusters also helps in the reduction of the noise and getting a better understanding of the genes involved in the clusters. Figure 8 shows the clusters generated by MCODE. The nodes in red color represent the seed proteins, and the yellow nodes are the connectors. The seed proteins are the proteins that were reported earlier to get either upregulated or downregulated by the action of NNK and connector proteins are the proteins that are associated with the seed proteins in the transfer of information, but are not reported to have any direct relation with NNK. The seed proteins were checked in all the clusters and are presented in bold in Table 1, while the remaining proteins (non-highlighted) are the connectors. The cluster that was ranked first had the highest score of 29,862, with 30 nodes and 433 edges. Moreover, during analysis, it was found that cluster 1 had 19 seed proteins and 11 connectors and the overall analysis of the entire cluster found 115 seeds and 88 connectors.

The seed proteins were identified in each cluster. Thereafter, the PPIN was generated using the STRING database with 0.9 confidence level, where 50 interactors in the first and another 50 in the second shell were recorded. The PPIN generated now was further enriched using the ClueGO plug-in.

### 3.4. PIN Construction and Topological and GO Analysis of Final Selected Seed Proteins

**Figure 9:**
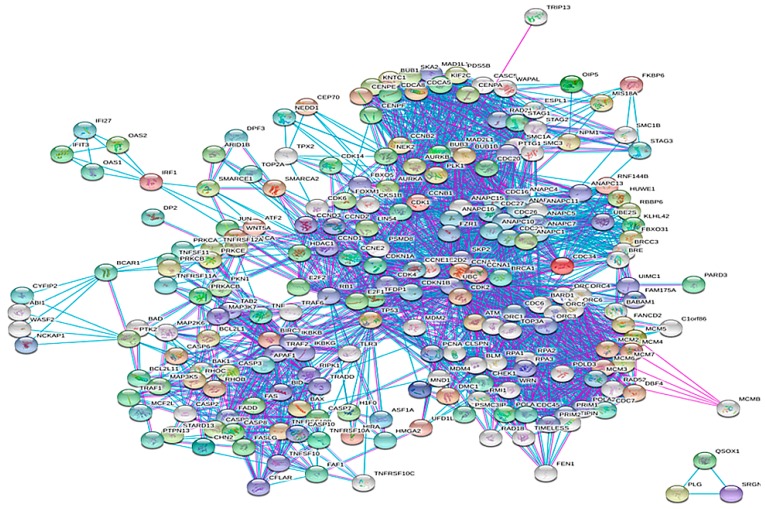
Protein–protein interaction network (PPIN) of final seed proteins.

After the modularization process, 115 seed proteins were obtained, and used to further create a PPIN (Figure 9) with 100 connectors using the highest confidence level score of 0.9. The PPIN generated had 213 nodes and 2509 edges, with an average node degree 23.6 and average clustering coefficient of 0.761. The PPI enrichment *p*-value was less than 1 × 10^−16^.

**Figure 10:**
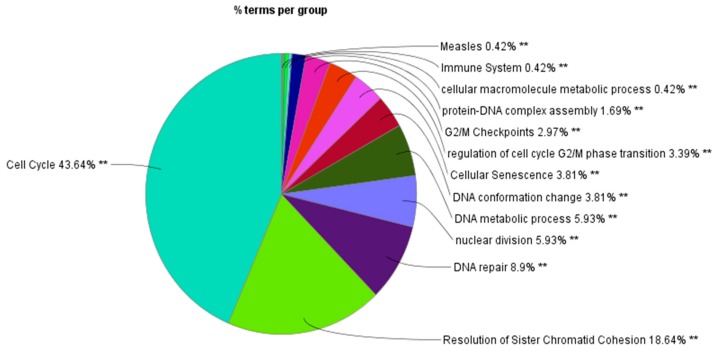
ClueGO results of gene ontology (GO) functional enrichment of key proteins.

**Figure 11:**
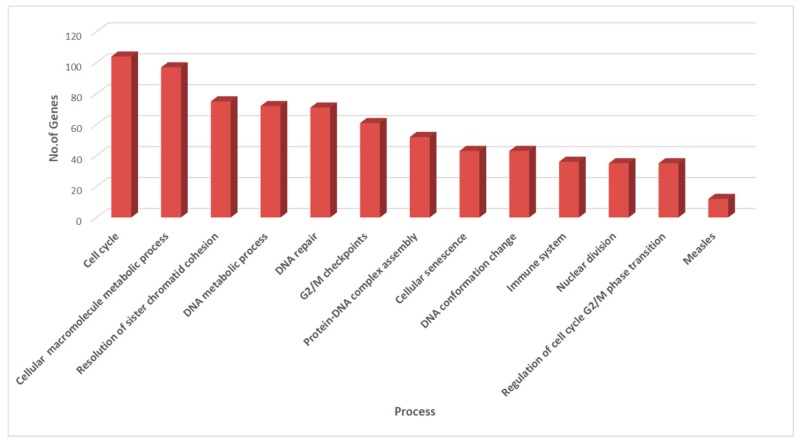
Representation of the pathways enriched and the total number of genes associated with them.

The network generated was analyzed using Cytohubba, a plug-in of Cytoscape [27]. Moreover, cytohubba analysis of each node of the network was performed and scored based on various parameters, like the degree, closeness centrality, clustering coefficients, betweenness, bottleneck, and stress. The proteins were first sorted on the basis of the clustering coefficient and then on the basis of bottleneck. The proteins with a clustering coefficient less than 0.5 were selected. The proteins having a clustering coefficient more than 0.5 were rejected as this depicts that these proteins were highly clustered and have no further spaces for the attachment of other molecules. The nodes with a clustering coefficient less than 0.5 and high degrees were found to be highly significant. A high degree and clustering coefficient less than 0.5 depict that the nodes are important in various connecting networks and they also have binding spaces available on their surface for other molecules to bind to. Once the nodes were sorted on the basis of clustering coefficients, the next most important parameter was the bottleneck. The proteins with high bottleneck were considered the most critical proteins in any PPIN. The median of the bottleneck scores of selected proteins was calculated which came out to be 3. All the proteins with bottleneck more than or equal to 2 were finally selected. Table 2 enlists the final selected proteins, which were sorted on the basis of bottleneck, with clustering coefficients less than 0.5. CHEK1, showing the highest bottleneck score of 29, was ranked in first position followed by TP53 with a bottleneck of 27. These were also analyzed with ClueGO for the pathway enrichment analysis. Figure 10 shows the pie chart representation of the enriched pathways in the form of groups. In Figure 10, the cell cycle is occupying the maximum area (43.64%) of the pie chart. From this, we can say that the cell cycle is the most enriched pathway, having the maximum number of biomolecules (a detailed graph displaying the sub-pathways in each group along with the number of genes present in it and the proteins involved in each group is enlisted in Table 3, also depicted in Supplementary Figure S1). Figure 11 shows that the maximum number of genes are associated with the cell cycle followed by the cellular macromolecule metabolic process.

### 3.5. Molecular Docking Simulation of NNK with Key Proteins

**Figure 12:**
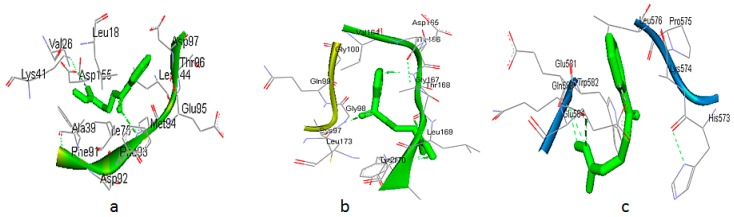
Binding interactions of the top three proteins with NNK: (**a**) CDK7 (−5.93 Kcal/Mol), (**b**) CCNA1 (−5.6 Kcal/Mol), (**c**) CDKN1B (−5.42 Kcal/Mol).

Autodock 4.0 was used for docking the final 30 target proteins with NNK. CDK7 showed the highest binding energy of −5.93 Kcal/Mol followed by CCNA1 (−5.6 Kcal/Mol). The binding energies of the proteins with NNK will further help in refining the results in the selection of the best-suited target proteins of NNK. The more negative the binding energy, the stronger the interaction between the ligand and the protein. The binding energies, Ki values, and the H-bonds formed along with their distances for all the 20 target proteins are listed in Table 4. Figure 12 shows the top three interactions of NNK with its target biomolecules, namely CDK7, CCNA1, and CDKN1B. Table 5 shows the key biomolecular targets of NNK and their role in cell cycle regulation.

## 4. Discussion

Cancer is a global inflammable problem, which pathologically occurs due to the accumulation of mutations in one or many genes, a phenomenon known as “oncogene addiction”. The loss of fidelity during the replication of DNA or the repair of damaged DNA leads to a cell becoming cancerous. Tobacco consumption has a major influence on these aberrations due to its main component, NNK, which has been strongly related with various cancers, mainly lung cancer [53,54]. NNK binds with various proteins involved in several cellular processes and leads to cancer. Hence, it is vital to fully understand the precise mechanism of the disease development caused by NNK and the interactome analysis of biomolecular target genes/proteins for attaining an overview of the highly complex functional interdependency of these targets for the efficient performance of the cell.

Systems biology is an interdisciplinary field that facilitates an understanding of complex biological network systems. These networks can be metabolic networks, gene regulation network, cell signaling network, or protein–protein interaction network [55]. With the help of system biology, we can analyze huge real-time networks precisely. Significantly, the huge amount of data from research being conducted globally were collected and statistically analyzed to uncover the functions of individual genes and proteins. The results may then be integrated to provide higher-level information [56]. Mathematical modeling and computational simulations help in understanding the internal dynamics of the system or the process and thus help in predicting the future of the process [57]. The networks created represent the genes or proteins with the nodes and their relations or physical connections with the edges [58]. The biological systems are immensely huge and complex networks depicting how one protein is connected with other proteins and how a slight impact on a gene or protein will affect the whole interactome [59]. These highly complicated and tightly packed networks, which govern all biological processes, are also referred to as protein–protein interaction networks (PPINs). All these graphs and networks work on the principle of graph theory. In the current study, we focused on NNK rewired PPIN to identify its bio-molecular targets that regulate the various cellular processes and cause cancer.

To construct PPIN, a literature survey was performed on PubMed database using keywords, like NNK, and around 544 genes extracted from 1317 research articles. The STRING database was used for the generation of the PPIN as it gives freedom to the users to choose the active interaction sources for their network. For this study, we used experimental data sources, like BIND, IntAct, etc., and pathway database interaction sources, like KEGG and reactome. The network generated had 534 nodes, including 100 connectors, at the highest confidence level score of 0.9. It contained 2909 edges that depicted that all the reported nodes were well connected with each other, which were further confirmed by the topological parameter, i.e., the average node degree, which was found to be 10.9. The degree of a node gives the information about the connection of the node with other nodes or simply the number of edges that either enter a node or leave it. All the biological systems are undirected and scale-free so the node degree depicts the average connectivity of each node with other nodes present in the network. The identification of key nodes in any network is not possible just by calculating its degree, hence various other parameters require evaluation, like the betweenness centrality, closeness centrality, and bottleneck scores [60]. Nodes with clustering coefficients less than 0.5 are key nodes because lesser clustering coefficients depict that the protein still have space for interaction with other molecules. The average neighborhood connectivity details provide the information about the density of the network. The shortest path length is important as it gives details about the minimum number of the edges between the two nodes and how fast information can pass on from one node to another node.

The network generated was then divided into modules. Modularization is an important step as it helps in reducing the noise of the data. On modularization, 19 clusters were obtained and each cluster contained seed proteins. Finally, 115 seed proteins were procured from the initial list of 534 proteins. Again, a PPIN was generated using these 115 seed proteins and 100 connectors at the first and second shells, and we obtained 213 nodes and 2509 edges. In addition, 100 connectors were done in order to include some more relevant proteins, which were possibly missed in the above procedure. The topological analysis was done on the basis of clustering coefficient and bottleneck scores. We used the bottleneck score as our final selection criteria as bottlenecks are the significant proteins, which have great functional and dynamic significance in a network [61]. To find the statistically significant cut-off for the proteins to be selected as key targets, the median was calculated on the bottleneck score, which came out to be 2. The enrichment analysis was done to find the pathways and processes that are enriched in the process of the PPIN generation. On the GO enrichment analysis, it was found that most of the seed proteins fall under the pathways that are related to cell cycle regulation and these results were also reflected on the final selected seed proteins that had a bottleneck score of 2 or more than 2. The cell cycle is a tightly regulated and a precisely timed event that is divided into phases and is monitored by checkpoints [62]. It is a highly orchestrated event, which ensures the integrity and stability of the genome. NNK binds to various receptors and hampers various pathways, leading to the cell becoming cancerous [63]. It not only binds with the receptors but also targets many important genes and proteins, like CDKs and cyclins, that are crucial for cell cycle processes [6]. Table 3 and Figure 10, Supplementary Figure S1, and Figure 11 clearly depict that most of the pathways that were enriched fall under cell cycle regulation at different phases.

Finally, docking studies were performed for the target proteins using NNK as a ligand. Molecular docking is the key tool to check whether the binding of ligand and protein is thermodynamically possible or not. The binding energies of these docking results further helped in refining the targets for NNK. The major targets of NNK found were CDK7, CCNA1, CDKN1B, CASP8, CHEK2, PLK1, BID, HSP90AA1, BRCA1, CDK1, CDK2, CCNB1, CHEK1, RPA2, ATM, CDK4, TFDP1, TP53, RB1, and RPA1, with their binding energies ranging from −5.93 to −3.67 Kcal/Mol. PYCARD and QXOS1 were excluded from this screening because of their low degree and 0 clustering coefficient. In addition to the above key proteins, our present study also revealed five connector proteins, which can be proven as putative biomolecular targets of NNK. These proteins are CHEK2, CCNA1, BID, RPA1, and RPA2. From these docking results, we can easily conclude that a majority of the key targets of NNK belong to the cell cycle regulatory proteome. CDKs and cyclins are the key regulatory proteins of the cell cycle that control the various phases of the cell. Different CDK/cyclin complexes are required for a smooth transition of the cell cycle. Other proteins, like ATM, RB1, and TP53, may act as tumor suppressor proteins and signaling proteins for DNA damage. Table 5 enlists the final key biomolecular targets of NNK and their role in the cell cycle.

## 5. Conclusions

In conclusion, a NNK rewired PPIN, with the help of various systems biology tools, was explored for the identification of potential targets involved in tobacco induced cancer development. The biomolecules that were suspected for being the most probable targets of NNK were further screened using functional enrichment techniques and then using molecular docking techniques. The results of this study show that the maximum proteins that are the most probable targets of NNK are involved in cell cycle regulation. With these, we can conclude that NNK has a major impact on the biomolecules of the cell cycle regulatory proteome. The present study opens future possibilities to identify new biomarkers for the cancers associated with NNK. This can also help in the pre-symptomatic diagnosis of diseases and in the development of precision medicines.

## Figures and Tables

**Table 1:** Generated clusters with MCODE scores, seeds (bold letters), and connector (non-bold letters) proteins.

Cluster	Score	Nodes	Edges	Seeds	Connectors	Node IDs
1	29.862	30	433	19	11	**PLK1**, CASC5, **BUB3**, **CDCA8**, **STAG1**, **BUB1B**, **CCNB2**, **CCNB1**, **BUB1**, ESPL1, **CENPF**, **KIF2C**, **CDCA5**, MAD2L1, **CDK1**, **PDS5B**, WAPAL, APITD1, **SKA2**, RAD21, SMC1A, **KNTC1**, **CDC20**, **CENPA**, CENPE, SMC3, STAG2, MAD1L1, **AURKB**, INCENP
2	19.3	41	386	22	19	**CDKN1A**, **FBXO31**, **CDC6**, **CDK2**, **RNF144B**, KLHL42, **CCNA1**, **DBF4**, **CDC7**, **MCM4**, POLA2, PRIM2, **CDC34**, **POLA1**, SKP2, ORC3, **MCM2**, **MCM5**, ORC2, ORC6, MCM6, MCM3, ORC4, **CHEK1**, **AURKA**, PTTG1, **CDC45**, PRIM1, MCM10, CDT1, ORC1, **HUWE1**, ORC5, **RBBP6**, **NEK2**, **MCM7**, ANAPC16, ANAPC13, **CDKN1B**, RPA1, **CCNA2**
3	14.652	47	337	9	38	ERCC2, **E2F1**, **FBXO5**, CDC23, ANAPC5, FANCE, **FANCC**, FANCG, BLM, ANAPC4, **ANAPC1**, **BRCA1**, **RB1**, FANCF, FANCL, C17orf70, CCNE1, C19orf40, **FANCD2**, FANCI, ANAPC10, FANCM, FANCB, GTF2H5, CCND2, FZR1, CDC26, RMI1, TOP3A, FANCA, GTF2H4, ANAPC11, CDC27, ANAPC2, GTF2H2, ANAPC7, GTF2H1, STRA13, CDC16, CCNH, C1orf86, ERCC3, **ATM**, CCND3, **BARD1**, GTF2H3, CCND1
4	10.762	22	113	13	9	**FAS**, RIPK1, **CASP8**, IKBKG, BID, **HDAC1**, **TNFRSF10B**, TRADD, **TP53**, **TFDP1**, TRAF2, TNFSF10, FADD, **FASLG**, **TNFRSF10A**, CASP10, **E2F2**, **TOPBP1**, IKBKB, **CDK4**, **CCNE2**, **CDK6**
5	7	7	21	7	0	**BBC3**, **BCL2A1**, **BCL2L11**, **BCL2**, **MCL1**, **PMAIP1**, **BCL2L1**
6	5	5	10	2	3	IGFBP1, **LGALS1**, IGFBP4, **QSOX1**, IGFBP5
7	4.333	7	13	5	2	NCKAP1, WASF2, **CYFIP2**, **PRKCA**, **PTK2**, **PRKCB**, **BCAR1**
8	4	4	6	4	0	**H1F0**, **HMGA2**, **ASF1A**, **HIRA**
9	4	4	6	1	3	KIAA1429, **WTAP**, CBLL1, ZC3H13
10	3.333	4	5	3	1	**EAF2**, GTF2H2C, **CDK7**, **MNAT1**
11	3.333	4	5	4	0	**OAS1**, **IFI27**, **OAS2**, **IFIT3**
12	3.333	7	10	5	2	**STAG3**, **CEP70**, **FKBP6**, SMC1B, **NEDD1**, **HSP90AA1**, TPX2
13	3	3	3	3	0	**MAP3K5**, **TRAF1**, **BIRC3**
14	3	3	3	3	0	**PSMC3IP**, **MND1**, **DMC1**
15	3	3	3	3	0	**OIP5**, **MIS18A**, **NPM1**
16	3	3	3	3	0	**TNFRSF11A**, **TNFRSF12A**, **TNFSF11**
17	3	3	3	3	0	**RHOB**, **RHOC**, **CDC25C**
18	3	3	3	3	0	**SDCBP**, **PYCARD**, **CPPED1**
19	3	3	3	3	0	**FGF2**, **FGFR3**, **FGF9**

Nodes in “bold text” represent the seed proteins and the nodes in “normal” text represent the connector proteins.

**Table 2:** Key proteins with their bottleneck, clustering coefficient, and degree scores.

Name	Betweenness	Bottleneck	Closeness	Clustering Coefficient	Degree
CHEK1	835.45994	29	116.61667	0.36501	52
TP53	8007.14223	27	126.41667	0.19394	55
BRCA1	2686.48081	23	127.45	0.26346	65
CDK1	3705.28949	19	140.41667	0.32157	85
CDK4	935.26901	14	112.66667	0.42521	35
HSP90AA1	3657.32532	13	101.66667	0.27273	22
RPA2	1523.40238	9	125.78333	0.33978	64
ATM	1803.73545	8	115.36667	0.33718	40
TFDP1	426.33754	6	111.78333	0.46702	34
CDKN1B	1218.04331	4	120.41667	0.44245	50
CASP8	1842.98595	4	90.11667	0.44853	17
PYCARD	796	3	62.18333	0.33333	3
CCNA1	894.78122	3	125.2	0.39548	60
CCNB1	1208.39103	3	127.26667	0.40665	69
RPA1	1657.28981	2	126.45	0.33269	65
CDK2	1887.40725	2	134.2	0.34035	76
CHEK2	126.13416	2	95.95	0.3619	15
BID	577.03969	2	90.78333	0.4269	19
RB1	608.84911	2	107.66667	0.44138	30
PLK1	1284.13385	2	121.25	0.49545	56
CDK7	488.15015	2	108.78333	0.49733	34

**Table 3:** Pathway enrichment of the modulated seed proteins using ClueGO.

Function	Groups	Group Genes
Cell cycle	Group12	ANAPC1|ASF1A|ATM|AURKA|AURKB|BARD1|BBC3|BCAR1|BCL2|BCL2A1|BCL2L1|BCL2L11| BIRC3|BRCA1|BUB1|BUB1B|BUB3|CASP8|CCNA1|CCNA2|CCNB1|CCNB2|CCNE2|CDC20|CDC25C| CDC34|CDC45|CDC6|CDC7|CDCA5|CDCA8|CDK1|CDK2|CDK6|CDKN1A|CENPA|CENPE|CENPF|CEP70|CHEK1|CYFIP2|DBF4|DMC1|E2F1|E2F2|EAF2|FANCC|FANCD2|FAS|FASLG|FBXO31|FBXO5|FGF2| FGF9|FGFR3|FKBP6|H1F0|HDAC1|HIRA|HMGA2|HSP90AA1|HUWE1|KIF2C|KNTC1|LGALS1|MCL1| MCM2|MCM4|MCM5|MCM6|MCM7|MDM2|MIS18A|MNAT1|MND1|NEDD1|NEK2|NPM1|OIP5| PDS5B|PLK1|PMAIP1|POLA1|PRKCA|PRKCB|PSMC3IP|PTK2|PYCARD|RBBP6|RHOB|RHOC|RNF144B|SDCBP|SKA2|STAG1|STAG3|TFDP1|TNFRSF10A|TNFRSF10B|TNFRSF10C|TNFRSF12A|TOPBP1|TP53|TRAF1
Cellular senescence	Group06	ANAPC1|ASF1A|ATM|BBC3|BCL2|BCL2L1|BCL2L11|BIRC3|BRCA1|CASP8|CCNA1| CCNA2|CCNE2|CDC20|CDC25C|CDCA5|CDK1|CDK2|CDK6|CDKN1A|CHEK1|E2F1|E2F2|FAS| FASLG|FGF2|FGF9|FGFR3|H1F0|HDAC1|HIRA|HMGA2|HSP90AA1|MAP3K5|MCL1|MDM2|PMAIP1| PRKCA|PRKCB|PTK2|TFDP1|TP53|TRAF1
DNA conformation change	Group07	ASF1A|ATM|BBC3|BCL2L11|BIRC3|CASP8|CCNB1|CDC45|CDCA5|CDK1|CENPA|CENPE|CENPF| DMC1|FANCC|FAS|FBXO5|H1F0|HDAC1|HIRA|HMGA2|HSP90AA1|KNTC1|MCM2|MCM4|MCM5| MCM6|MCM7|MDM2|MIS18A|MNAT1|NPM1|OAS1|OIP5|PMAIP1|POLA1|PRKCA|PTK2|PYCARD| RHOC|TNFSF11|TP53|TRAF1
DNA metabolic process	Group08	ASF1A|ATM|AURKA|AURKB|BARD1|BBC3|BCL2|BCL2A1|BCL2L1|BCL2L11|BRCA1|BUB1|BUB1B|BUB3|CCNA1|CCNA2|CCNB1|CCNE2|CDC25C|CDC34|CDC45|CDC6|CDC7|CDCA5|CDK1|CDK2|CDK6|CDKN1A|CENPF|CHEK1|DBF4|DMC1|E2F1|EAF2|FANCC|FANCD2|FAS|FBXO31|FBXO5|FKBP6|H1F0|HDAC1|HMGA2|HSP90AA1|HUWE1|KNTC1|MCL1|MCM2|MCM4|MCM5|MCM6|MCM7|MDM2|MIS18A|MNAT1|MND1|NEK2|NPM1|PDS5B|PLK1|PMAIP1|POLA1|PRKCB|PSMC3IP|PYCARD|RBBP6|TFDP1|TNFRSF10A|TNFRSF10B|TNFRSF10C|TOPBP1|TP53
DNA repair	Group10	ANAPC1|ASF1A|ATM|AURKA|AURKB|BARD1|BBC3|BCL2|BCL2L11|BRCA1|CASP8|CCNA1|CCNA2| CCNB1|CCNB2|CCNE2|CDC20|CDC25C|CDC34|CDC45|CDC6|CDC7|CDCA5|CDK1|CDK2|CDK6|CDKN1A|CENPF|CEP70|CHEK1|DBF4|DMC1|E2F1|E2F2|EAF2|FANCC|FANCD2|FAS|FASLG|H1F0|HDAC1| HIRA|HMGA2|HSP90AA1|HUWE1|MAP3K5|MCM2|MCM4|MCM5|MCM6|MCM7|MDM2|MNAT1| MND1|NEDD1|NEK2|NPM1|PDS5B|PLK1|PMAIP1|POLA1|PRKCA|PRKCB|RBBP6|TFDP1|TNFRSF10A|TNFRSF10B|TNFRSF10C|TOPBP1|TP53|TRAF1
G2/M checkpoints	Group04	ASF1A|ATM|AURKA|AURKB|BARD1|BBC3|BCL2|BCL2A1|BCL2L1|BCL2L11|BRCA1|CCNA1|CCNA2| CCNB1|CCNB2|CCNE2|CDC25C|CDC34|CDC45|CDC6|CDC7|CDCA5|CDK1|CDK2|CDKN1A|CENPF| CHEK1|DBF4|DMC1|E2F1|FANCC|FANCD2|FBXO31|FKBP6|H1F0|HDAC1|HMGA2|HSP90AA1|HUWE1|MCL1|MCM2|MCM4|MCM5|MCM6|MCM7|MDM2|MIS18A|MNAT1|MND1|NEK2|NPM1|PDS5B|PLK1|PMAIP1|POLA1|PSMC3IP|PYCARD|RBBP6|TFDP1|TOPBP1|TP53
Immune system	Group01	ANAPC1|BCL2|BCL2L1|BIRC3|CASP8|CDC20|CDC34|CDK1|CDKN1A|CENPE|CPPED1|CYFIP2|FASLG| FBXO31|FGF2|FGF9|FGFR3|HSP90AA1|HUWE1|IFI27|IFIT3|KIF2C|MCL1|OAS1|OAS2|PRKCB|PTK2| PYCARD|QSOX1|RBBP6|RNF144B|SDCBP|TNFRSF11A|TNFRSF12A|TNFSF11|TP53
Measles	Group00	BBC3|CCNE2|CDK2|CDK6|FAS|FASLG|OAS1|OAS2|TNFRSF10A|TNFRSF10B|TNFRSF10C|TP53
Resolution of sister chromatid cohesion	Group11	ANAPC1|ATM|AURKA|AURKB|BARD1|BBC3|BCL2|BCL2L11|BIRC3|BRCA1|BUB1|BUB1B|BUB3|CASP8|CCNA1|CCNA2|CCNB1|CCNB2|CCNE2|CDC20|CDC25C|CDC34|CDC6|CDC7|CDCA5|CDCA8|CDK1|CDK2|CDKN1A|CENPA|CENPE|CENPF|CEP70|CHEK1|CYFIP2|DMC1|E2F1|FANCD2|FAS|FASLG|FBXO31|FBXO5|FKBP6|HMGA2|HSP90AA1|HUWE1|IFI27|KIF2C|KNTC1|MAP3K5|MDM2|MNAT1|MND1|NEDD1|NEK2|NPM1|PDS5B|PLK1|PMAIP1|PRKCA|PRKCB|PSMC3IP|PTK2|PYCARD|RBBP6|RNF144B|SDCBP|SKA2|STAG1|STAG3|TFDP1|TNFRSF10A|TNFRSF10B|TOPBP1|TP53
Cellular macromolecule metabolic process	Group02	ANAPC1|ASF1A|ATM|AURKA|AURKB|BARD1|BBC3|BCL2|BCL2L11|BIRC3|BRCA1|BUB1|BUB1B|BUB3|CASP8|CCNA1|CCNA2|CCNB1|CCNE2|CDC20|CDC25C|CDC34|CDC45|CDC6|CDC7|CDCA5|CDCA8|CDK1|CDK2|CDK6|CDKN1A|CENPE|CENPF|CHEK1|CPPED1|CYFIP2|DBF4|DMC1|E2F1|E2F2|EAF2|FANCC|FANCD2|FAS|FASLG|FBXO31|FBXO5|FGF2|FGF9|FGFR3|FKBP6|H1F0|HDAC1|HIRA|HMGA2|HSP90AA1|HUWE1|IFI27|LGALS1|MAP3K5|MCM2|MCM4|MCM5|MCM6|MCM7|MDM2|MIS18A|MNAT1|MND1|NEK2|NPM1|OAS1|OAS2|PDS5B|PLK1|PMAIP1|POLA1|PRKCA|PRKCB|PSMC3IP|PTK2|PYCARD|QSOX1|RBBP6|RNF144B|SDCBP|STAG1|TFDP1|TNFRSF10A|TNFRSF10B|TNFRSF10C|TNFRSF11A|TNFSF11|TOPBP1|TP53|TRAF1|WTAP
Nuclear division	Group09	ANAPC1|ATM|AURKA|AURKB|BRCA1|BUB1|BUB1B|BUB3|CCNA1|CCNA2|CCNB1|CCNE2|CDC20| CDC25C|CDC6|CDCA5|CDCA8|CDK1|CDK2|CENPE|CENPF|CHEK1|DMC1|FANCD2|FBXO5|FKBP6| KIF2C|KNTC1|MND1|NEK2|PDS5B|PLK1|PSMC3IP|STAG1|STAG3
Protein–DNA complex assembly	Group03	ASF1A|ATM|AURKA|AURKB|BBC3|BCL2|BCL2L11|BIRC3|BRCA1|CASP8|CCNB1|CDC20|CDC34|CDC45|CDK1|CDK2|CENPA|CENPE|CENPF|CEP70|DMC1|FANCC|FAS|FBXO5|H1F0|HDAC1|HIRA|HMGA2|HSP90AA1|KNTC1|MCM2|MCM7|MDM2|MIS18A|MNAT1|NEDD1|NEK2|NPM1|OAS1|OIP5|PLK1|PMAIP1|PRKCA|PTK2|PYCARD|RHOC|SDCBP|STAG1|STAG3|TNFSF11|TP53|TRAF1
Regulation of cell cycle G2/M phase transition	Group05	ANAPC1|ATM|AURKA|AURKB|BARD1|BRCA1|BUB1B|BUB3|CCNA1|CCNA2|CCNB1|CCNB2|CDC20| CDC25C|CDC7|CDK1|CDK2|CDK6|CDKN1A|CENPF|CEP70|CHEK1|FBXO5|HMGA2|HSP90AA1|MDM2|MNAT1|NEDD1|NEK2|NPM1|PLK1|PRKCA|PRKCB|TOPBP1|TP53

**Table 4:** Final 22 target proteins docked with NNK.

S.No	Protein	Ligand	Binding Energy (Kcal/Mol)	K_i_	Binding Residues	H-Bond	Distance
1.	CDK7	NNK	−5.93	45.31 μM	Leu18, Val26, Ala39, Lys41, Ile75, Phe91, Asp92, Phe93, Met94, Glu95, Thr96, Asp97, Leu144, Asp155	CDK7:MET94:N - :NNK:O7	2.86224
2.	CCNA1 (connector)	NNK	−5.60	79.09 μM	Cys97, Gly98, Gln99, Gly100, Val164, Asp165, Thr166, Gly167, Thr168, Leu169, Lys170, Leu173, Tyr218	:GLY98:H - :NNK:O7 :GLY167:H - :NNK:N10 :THR168:H - :ASP165:O :LYS170:H - :NNK:N14 :LYS170:H - :NNK:O15 :NNK:N14 - :THR168:O	1.83311 1.98057 2.11393 2.36928 1.99807 3.02246
3.	CDKN1B	NNK	−5.42	106.27 μM	His573, Lys574, Pro575, Leu576, Glu581, Trp582, Gln583, Glu584	CDKN1B:GLN583:N - :NNK:N14 CDKN1B:GLN583:N - :NNK:O15 :NNK:N14 - CDKN1B:GLN583:O	2.84311 2.79833 2.96186
4.	CASP8	NNK	−5.35	119.75 μM	Lys2224, Tyr2226, Gln2227, Asp2308, Gly2350, Lys2351, Pro2352, Asp2398, Arg2471, Lys2472	CASP8:LYS2351:HZ1 - :NNK:N10 CASP8:ARG2471:HH21 - :NNK:N2 CASP8:ARG2471:HH21 - :NNK:N14 CASP8:ARG2471:HH21 - :NNK:O15 CASP8:LYS2472:HZ1 - :NNK:O7	1.9527 2.48624 2.19123 2.25672 1.82255
5.	CHEK2 (connector)	NNK	−5.35	120.25 μM	Ser228, Gly229, Ala230, Cys231, Gly232, Val234, Lys249, Leu301, Thr367, Asp368	CHEK2:CYS231:N - :NNK:N10 CHEK2:GLY232:N - :NNK:N10 CHEK2:LYS249:NZ - :NNK:N14 CHEK2:ASP368:N - :NNK:O15	3.11295 3.11412 3.20168 2.86381
6.	PLK1	NNK	−5.21	152.98 μM	Lys413, Trp414, Val415, Asp416, Leu490, Asn533, Lys540	PLK1:TRP414:N - :NNK:O7 PLK1:ASP416:N - :NNK:N14 PLK1:ASP416:N - :NNK:O15 PLK1:ASN533:ND2 - :NNK:N10	2.97424 2.94596 2.71702 3.10323
7.	BID (connector)	NNK	−5.13	174.27 μM	Leu21, Phe24, Gly25, Gln28, Leu39, Asp40, Leu42, Gly43, Arg86, Ala89, Arg90, Phe173	:NNK:N14 - BID:GLN28:OE1 BID:PHE24:HA - :NNK:O15 BID:ARG86:HA - :NNK:N10 :NNK:C3 - BID:GLN28:OE1 :NNK:C1 - BID:LEU39:O :NNK:C11 - BID:ARG86:O :NNK:O15 - BID:PHE24 :NNK:N14 - BID:GLN28:OE1 BID:PHE24:HA - :NNK:O15 BID:ARG86:HA - :NNK:N10 :NNK:C3 - BID:GLN28:OE1 :NNK:C1 - BID:LEU39:O :NNK:C11 - BID:ARG86:O :NNK:O15 - BID:PHE24	3.29101 2.94776 2.82375 3.41587 3.00214 3.32997 3.70686 3.29101 2.94776 2.82375 3.41587 3.00214 3.32997 3.70686
8.	HSP90AA1	NNK	−5.10	183.46 μM	Leu48, Asn51, Ser52, Ala55, Asp93, Ile96, Gly97, Met98, Asn106, Phe138, Thr184, Val186	HSP90AA1:ASN51:ND2 - :NNK:N14 HSP90AA1:ASN51:ND2 - :NNK:O15 HSP90AA1:THR184:OG1 - :NNK:O7	3.12471 2.99653 2.69827
9.	BRCA1	NNK	−5.08	187.68 μM	Val1654, Ser1655, Gly1656, Leu1657, Thr1658, Pro1659, Phe1662, Thr1700, Leu1701, Lys1702	BRCA1:SER1655:OG - :NNK:N14 BRCA1:GLY1656:N - :NNK:O7 BRCA1:LEU1657:N - :NNK:O7 BRCA1:LEU1701:N - :NNK:O15 BRCA1:LYS1702:N - :NNK:O15 :NNK:N14 - BRCA1:SER1655:OG	3.19252 2.75464 2.77131 3.00037 2.75018 3.19252
10.	CDK1	NNK	−5.00	217.06 μM	Lys88, Leu91, Asp92, Ile94, Pro95, Pro96, Glu196, Lys200	CDK1:LYS200:NZ - :NNK:N14 CDK1:LYS200:NZ - :NNK:O15 :NNK:N14 - CDK1:ILE94:O	3.0124 2.96559 2.89466
11.	CDK2	NNK	−4.90	255.02 μM	Val29, Glu81, Phe82, Leu83, His84, Ile135, Asn136, Thr137	CDK2:PHE82:N - :NNK:N10 CDK2:HIS84:N - :NNK:N14 CDK2:HIS84:N - :NNK:O15 CDK2:HIS84:ND1 - :NNK:N14 :NNK:O15 - CDK2:ILE135:O	2.88593 2.95506 2.76842 3.18956 2.91961
12.	CCNB1	NNK	−4.86	274.97 μM	Ile253, Lys256, Tyr257, Glu285, Leu289, Phe294, Gly295, Leu296, Gly297	CCNB1:TYR257:N - :NNK:N10 CCNB1:LEU296:N - : NNK:N14 CCNB1:LEU296:N - : NNK:O15 CCNB1:GLY297:N - : NNK:O15 : NNK:N14 - CCNB1:PHE294:O	3.14377 2.86319 2.81795 2.91169 3.10697
13.	CHEK1	NNK	−4.80	303.50 μM	Val23, Val37, Ile39, Glu55, Asn59, Leu82, Phe149	CHEK1:ILE39:N - :NNK:N10 CHEK1:ASN59:ND2 - :NNK:N14 CHEK1:ASN59:ND2 - :NNK:O15 CHEK1:PHE149:N - :NNK:O15 :NNK:N14 - CHEK1:GLU55:OE2 :NNK:O15 - CHEK1:GLU55:OE2 :NNK:O15 - CHEK1:PHE149:N	2.8265 2.71952 3.02158 3.15867 3.05383 2.67183 3.15867
14.	RPA2 (connector)	NNK	−4.70	358.90 μM	Cys49, Thr50, Ile76, Val77, Asp96, Met97, Tyr125, Phe155, His158, Ile159	RPA2:VAL77:N - :NNK:O15 :NNK:O15 - RPA2:CYS49:O :NNK:O15 - RPA2:VAL77:O :NNK:O15 - RPA2:HIS158:NE2	3.09031 3.06724 3.18087 2.98064
15.	ATM	NNK	−4.48	523.49 μM	Thr2059, Ala2062, Gly2063, Ile2065, Gln2066, Gln2069, Leu2077, Tyr2080, Leu2081, Leu2084, Glu2094, Leu2095, Leu2098	ATM:GLN2066:N - :NNK:O7	3.06063
16.	CDK4	NNK	−4.35	650.98 μM	Val44, Leu54, Pro55, Thr58, Val59, Val62, Ala63, Arg66, Val82, Ile92, Val94	CDK4:PRO55:CD - :NNK:O15 CDK4:VAL59:CA - :NNK:O7	2.9883 2.92285
17.	TFDP1	NNK	−4.20	833.96 μM	Val264, Phe285, Asn286, Phe287, Phe291	TFDP1:PHE287:N - :NNK:O15 :NNK:O15 - TFDP1:PHE287:O	2.78538 3.08744
18.	TP53	NNK	−4.14	927.72 μM	Gln136, Leu137, Ala138, His179, Cys182, Asp184, Asn239, Cys275, Ala276	TP53:LEU137:N - :NNK:O15 TP53:ASN239:ND2 - :NNK:N14 TP53:ASN239:ND2 - :NNK:O15 :NNK:O15 - TP53:CYS275:O	2.93164 3.07617 2.87469 2.82627
19.	RB1	NNK	−4.02	1.14 mM	Val434, Gly435, Gln436, Cys438, Asn505, Leu506, Asp507, Ser508, Gly509, Thr510	RB1:GLN436:HN - :NNK:O15 RB1:SER508:HN - :NNK:O7 RB1:GLY509:HN - :NNK:O7	1.93726 2.36625 1.90514
20.	RPA1 (connector)	NNK	−3.67	2.03 mM	Val375, Asn402, Pro403, Ala408, Tyr409, Arg412, Gly413	RPA1:ARG412:NH1 - :NNK:N14 :NNK:O15 - RPA1:ALA408:O	3.06161 2.78707

**Table 5:** Key proteins and their roles in cell cycle regulation.

S.No	Protein	Role in Cell Cycle	References
1.	CDK7	Catalytic subunit of CAK complex which activates the cyclin-associated kinases CDK1, CDK2, CDK4, and CDK6 by threonine phosphorylation, thus regulating cell cycle progression	[28,29]
2.	CCNA1	Controls the cell cycle at the G1/S (start) and G2/M (mitosis) transitions	[30]
3.	CDKN1B	Important regulator of cell cycle progression. Acts either as an inhibitor or an activator of cyclin type D-CDK4 complexes depending on its phosphorylation state and/or stoichometry.	[31]
4.	CASP8	Responsible for the positive and negative regulation of apoptosis and inflammation	[32]
5.	CHEK2	required for checkpoint-mediated cell cycle arrest in response to p53 defects	[33]
6.	PLK1	Regulates entry and exit from mitosis and cytokinesis. Regulates DNA replication	[34]
7.	BID	induces apoptosis on interaction with BCl2 family proteins	[35]
8.	HSP90AA1	regulates the function of ATM in sensing and repairing the DNA damages	[36]
9.	BRCA1	Has role in DNA damage repair, cell cycle control and transcriptional regulation	[37]
10.	CDK1	Promotes G2-M transition and regulates G1 progress and G1-S transition via association with multiple interphase cyclins.	[38]
11.	CDK2	Essential for transition of cell cycle from G1 to S phase and then from S to G2 phase.	[39,40]
12.	CCNB1	Essential for the control of the cell cycle at the G2/M (mitosis) transition	[41]
13.	CHEK1	required for smooth cellular proliferation by inducing the degradation of cdc25a	[42]
14.	RPA2	Binds and stabilizes single-stranded DNA intermediates. Controls DNA repair and DNA damage checkpoint activation.	[43,44]
15.	ATM	Activates checkpoint signaling upon double strand breaks (DSBs), apoptosis. May function as a tumor suppressor.	[45,46]
16.	CDK4	Hypophosphorylates RB1 in early G_1_ phase. Cyclin D-CDK4 complexes are major integrators of various mitogenic and antimitogenic signals.	[47]
17.	TFDP1	Can stimulate E2F-dependent transcription induces growth arrest.	[48]
18.	TP53	Prevents CDK7 kinase activity when associated to CAK complex in response to DNA damage, thus stopping cell cycle progression.	[49,50]
19.	RB1	Key regulator of entry into cell division that acts as a tumor suppressor. Promotes G0-G1 transition when phosphorylated by CDK3/cyclin-C.	[51]
20.	RPA1	controls DNA repair and DNA damage checkpoint activation	[52]

## Supplementary Materials

The following are available online at https://www.mdpi.com/2073-4425/10/8/564/s1, Figure S1: Graph of GO enriched pathways depicting the involvement of genes under various sub-graphs.
