# Supplementary material for: Investigation of Precise Molecular Mechanistic Action of Tobacco-Associated Carcinogen ‘NNK’ Induced Carcinogenesis: A System Biology Approach
Source: Genes (Basel). 2019 Jul 26;10(8):564. doi: 10.3390/genes10080564 (PMC6723528; doi:10.3390/genes10080564)
Supplement: Supplementary file 1 [file genes-10-00564-s001.pdf]

The supplementary figure 1, depicts the genes involved in various subgraphs.

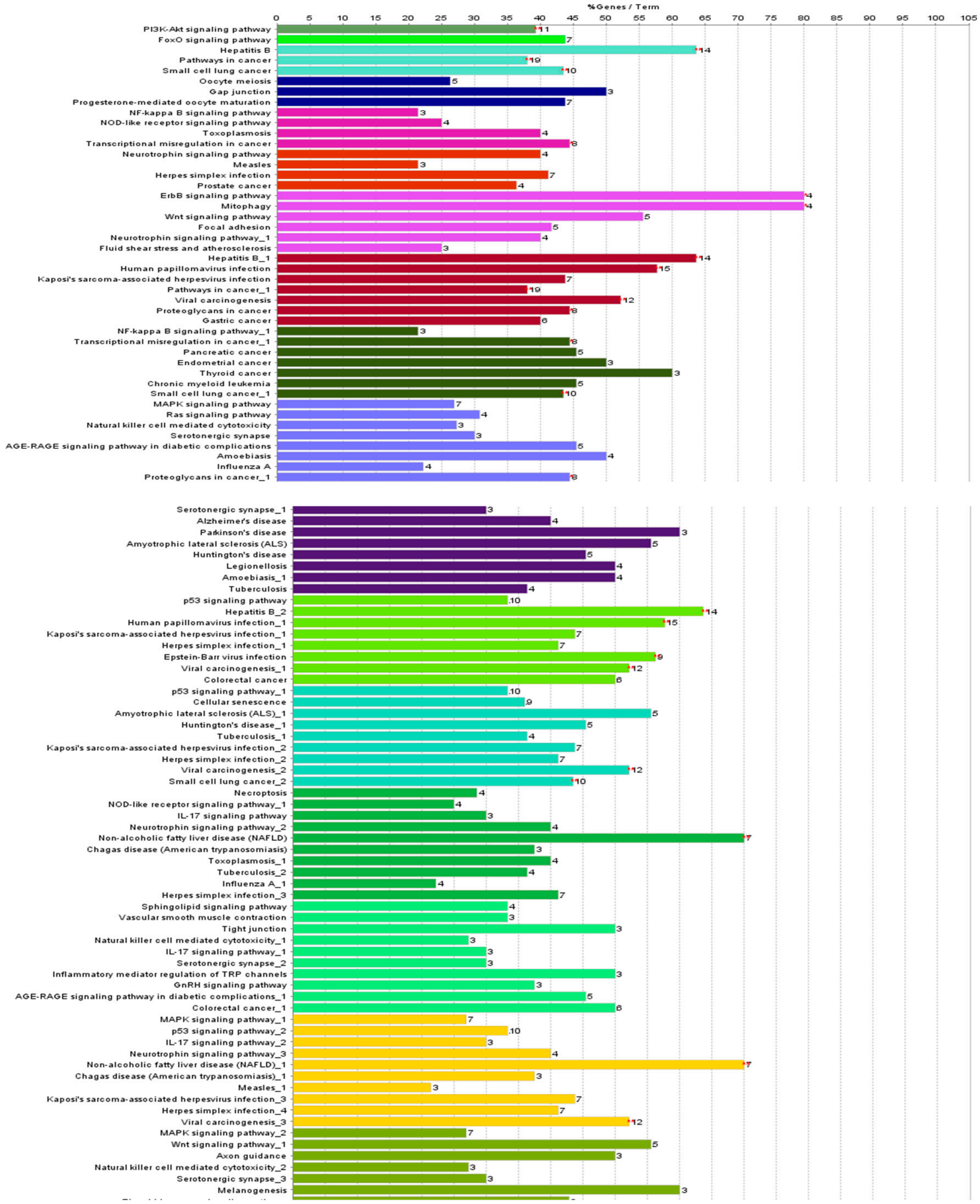

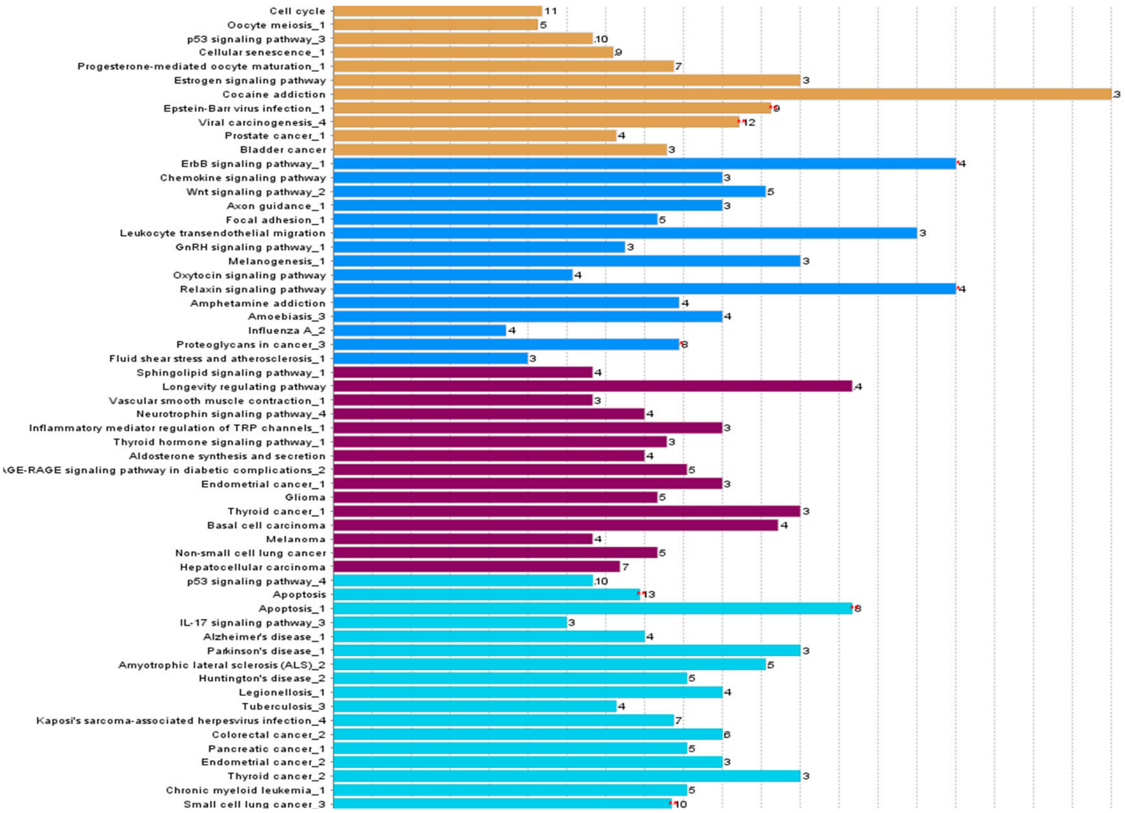
Cell cycle 11
Oocyte meiosis_1 5
p53 signaling pathway_3 10
Cellular senescence_1 9
Progesterone-mediated oocyte maturation_1 7
Estrogen signaling pathway 3
Cocaine addiction 3
Epstein-Barr virus infection_1 9
Viral carcinogenesis_4 12
Prostate cancer_1 4
Bladder cancer 3
ErbB signaling pathway_1 4
Chemokine signaling pathway 3
Wnt signaling pathway_2 5
Axon guidance_1 3
Focal adhesion_1 5
Leukocyte transendothelial migration 3
GnRH signaling pathway_1 3
Melanogenesis_1 3
Oxytocin signaling pathway 4
Relaxin signaling pathway 4
Amphetamine addiction 4
Amoebiasis_3 4
Influenza A_2 4
Proteoglycans in cancer_3 8
Fluid shear stress and atherosclerosis_1 3
Sphingolipid signaling pathway_1 4
Longevity regulating pathway 4
Vascular smooth muscle contraction_1 3
Neurotrophin signaling pathway_4 4
Inflammatory mediator regulation of TRP channels_1 3
Thyroid hormone signaling pathway_1 3
Aldosterone synthesis and secretion 4
GE-RAGE signaling pathway in diabetic complications_2 5
Endometrial cancer_1 3
Glioma 5
Thyroid cancer_1 3
Basal cell carcinoma 4
Melanoma 4
Non-small cell lung cancer 5
Hepatocellular carcinoma 7
p53 signaling pathway_4 10
Apoptosis 13
Apoptosis_1 8
IL-17 signaling pathway_3 3
Alzheimer's disease_1 4
Parkinson's disease_1 3
Amyotrophic lateral sclerosis (ALS)_2 5
Huntington's disease_2 5
Legionellosis_1 4
Tuberculosis_3 4
Kaposi's sarcoma-associated herpesvirus infection_4 7
Colorectal cancer_2 6
Pancreatic cancer_1 5
Endometrial cancer_2 3
Thyroid cancer_2 3
Chronic myeloid leukemia_1 5
Small cell lung cancer_3 10

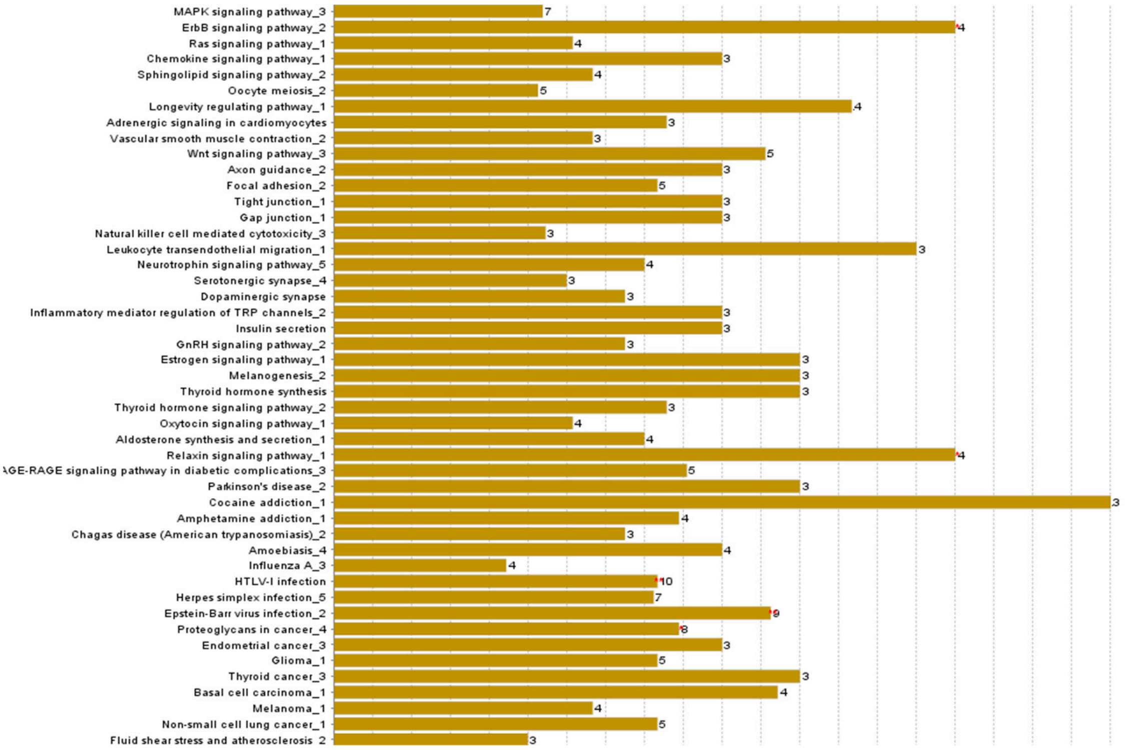
MAPK signaling pathway_3 7
ErbB signaling pathway_2 4
Ras signaling pathway_1 4
Chemokine signaling pathway_1 3
Sphingolipid signaling pathway_2 4
Oocyte meiosis_2 5
Longevity regulating pathway_1 4
Adrenergic signaling in cardiomyocytes 3
Vascular smooth muscle contraction_2 3
Wnt signaling pathway_3 5
Axon guidance_2 3
Focal adhesion_2 5
Tight junction_1 3
Gap junction_1 3
Natural killer cell mediated cytotoxicity_3 3
Leukocyte transendothelial migration_1 3
Neurotrophin signaling pathway_5 4
Serotonergic synapse_4 3
Dopaminergic synapse 3
Inflammatory mediator regulation of TRP channels_2 3
Insulin secretion 3
GnRH signaling pathway_2 3
Estrogen signaling pathway_1 3
Melanogenesis_2 3
Thyroid hormone synthesis 3
Thyroid hormone signaling pathway_2 3
Oxytocin signaling pathway_1 4
Aldosterone synthesis and secretion_1 4
Relaxin signaling pathway_1 4
GE-RAGE signaling pathway in diabetic complications_3 5
Parkinson's disease_2 3
Cocaine addiction_1 3
Amphetamine addiction_1 4
Chagas disease (American trypanosomiasis)_2 3
Amoebiasis_4 4
Influenza A_3 4
HTLV-I infection 10
Herpes simplex infection_5 7
Epstein-Barr virus infection_2 9
Proteoglycans in cancer_4 8
Endometrial cancer_3 3
Glioma_1 5
Thyroid cancer_3 3
Basal cell carcinoma_1 4
Melanoma_1 4
Non-small cell lung cancer_1 5
Fluid shear stress and atherosclerosis_2 3

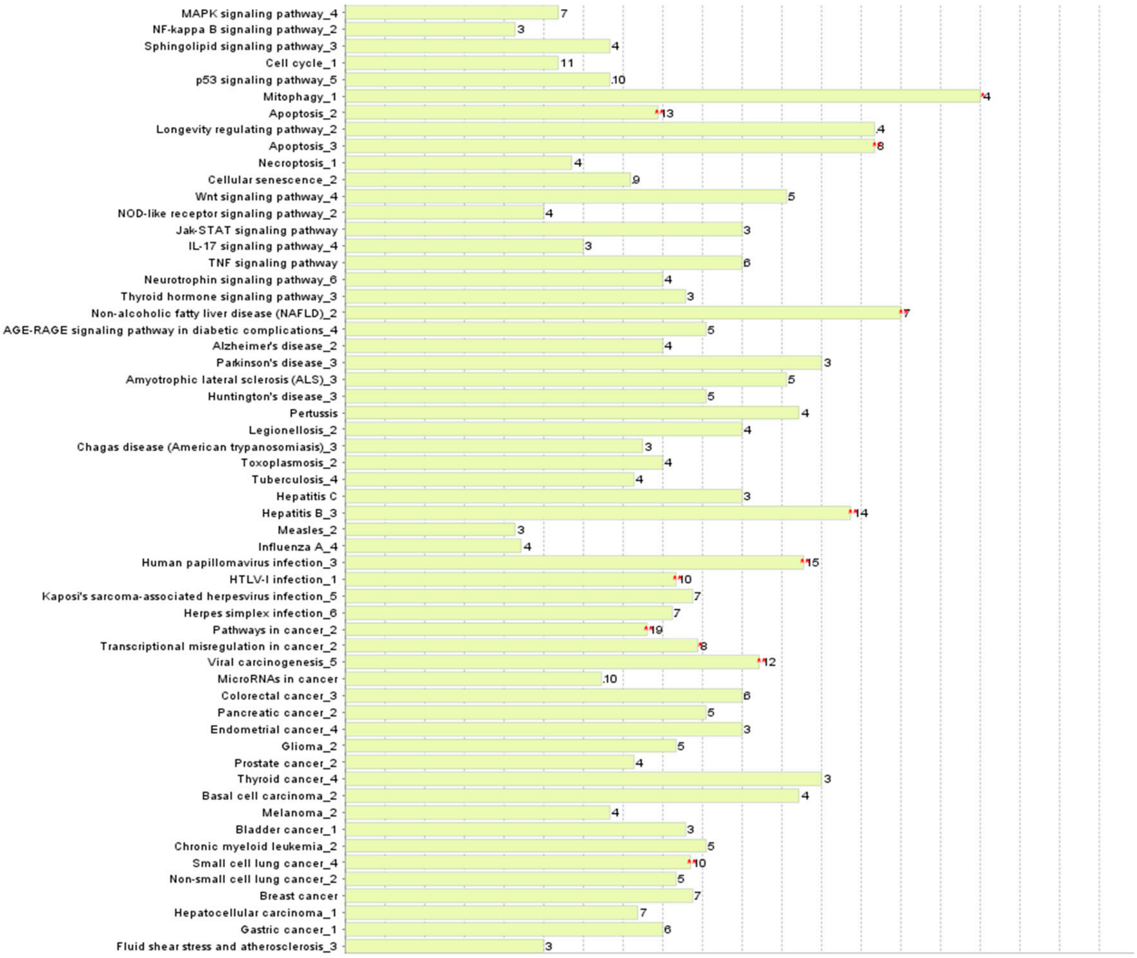

Supplementary Figure 1: Graph of GO enriched pathways depicting the involvement of genes under various sub-graphs. The x-axis showing the percent (%) of genes/biomolecules against pathway term and the y-axis depicting the various pathways that have got enriched. Same colored bars depict a group.
